# Diazepam Promotes Translocation of Human Constitutive Androstane Receptor (CAR) via Direct Interaction with the Ligand-Binding Domain

**DOI:** 10.3390/cells9122532

**Published:** 2020-11-24

**Authors:** Josef Skoda, Jan Dusek, Martin Drastik, Alzbeta Stefela, Klara Dohnalova, Karel Chalupsky, Tomas Smutny, Stanislav Micuda, Sabine Gerbal-Chaloin, Petr Pavek

**Affiliations:** 1 Department of Pharmacology and Toxicology, Faculty of Pharmacy, Charles University, Heyrovskeho 1203, 500 05 Hradec Kralove, Czech Republic; skodajo@faf.cuni.cz (J.S.); dusekja3@faf.cuni.cz (J.D.); stefelaa@faf.cuni.cz (A.S.); smutt6aa@faf.cuni.cz (T.S.); 2 Department of Physical Chemistry and Biophysics, Faculty of Pharmacy, Charles University, Heyrovskeho 1203, 500 05 Hradec Kralove, Czech Republic; drastikm@faf.cuni.cz; 3 1^st^ Medical Faculty, Charles University, Katerinská 32, 121 08 Prague, Czech Republic; klara.dohnalova@img.cas.cz; 4 Czech Centre for Phenogenomics, Institute of Molecular Genetics of the Czech Academy of Sciences, Vídeňská 1083, 142 20 Prague, Czech Republic; krlcha@img.cas.cz; 5 Department of Pharmacology, Medical Faculty in Hradec Kralove, Charles University, Simkova 870, 500 03 Hradec Kralove, Czech Republic; micuda@lfhk.cuni.cz; 6 IRMB, University of Montpellier, INSERM, 34295 Montpellier, France; sabine.gerbal@gmail.com

**Keywords:** diazepam, gene regulation, CAR, NR1I3, cytochrome P450, drug interaction

## Abstract

The constitutive androstane receptor (CAR) is the essential regulator of genes involved both in xenobiotic and endobiotic metabolism. Diazepam has been shown as a potent stimulator of CAR nuclear translocation and is assumed as an indirect CAR activator not interacting with the CAR cavity. In this study, we sought to determine if diazepam is a ligand directly interacting with the CAR ligand binding domain (LBD) and if it regulates its target genes in a therapeutically relevant concentration. We used different CAR constructs in translocation and luciferase reporter assays, recombinant CAR-LBD in a TR-FRET assay, and target genes induction studied in primary human hepatocytes (PHHs), HepaRG cells, and in CAR humanized mice. We also used in silico docking and CAR-LBD mutants to characterize the interaction of diazepam and its metabolites with the CAR cavity. Diazepam and its metabolites such as nordazepam, temazepam, and oxazepam are activators of CAR+Ala in translocation and two-hybrid assays and fit the CAR cavity in docking experiments. In gene reporter assays with CAR3 and in the TR-FRET assay, only diazepam significantly interacts with CAR-LBD. Diazepam also promotes up-regulation of CYP2B6 in PHHs and in HepaRG cells. However, in humanized CAR mice, diazepam significantly induces neither *CYP2B6* nor *Cyp2b10* genes nor does it regulate critical genes involved in glucose and lipids metabolism and liver proliferation. Thus, we demonstrate that diazepam interacts with human CAR-LBD as a weak ligand, but it does not significantly affect expression of tested CAR target genes in CAR humanized mice.

## 1. Introduction

The nuclear receptor constitutive androstane receptor (CAR, NR1I3) is primarily expressed in the liver and acts as an essential xenobiotic and endobiotic sensor. After xenobiotic stimulation, CAR drives the cellular response by modulating the expression of a wide set of drug metabolizing enzymes and transporters [1,2]. Besides xenobiotic detoxification, activation of CAR is also involved in the regulation of other hepatic functions such as gluconeogenesis, lipid synthesis, fatty acid oxidation, and clearance of steroid hormones and bilirubin [2,3].

CAR activation is a multi-step process with the nuclear accumulation of CAR monomer being the first key event. The interaction of the CAR/RXRα heterodimer with the regulatory elements of target genes subsequently triggers a cellular response. CAR is activated either by ligand interaction with the CAR ligand-binding domain (CAR-LBD) or indirectly by dephosphorylation via the signaling of the epidermal growth factor (EGF) [4,5,6].

Diazepam was identified as an indirect activator of human CAR (hCAR) in a translocation assay in primary human hepatocytes [7]. In rodents, diazepam is a CYP2B inducer and promotes liver tumorigenesis as a non-genotoxic carcinogen [8]. However, the exact mechanism of CAR activation mediated by diazepam remains unrevealed. In addition, neither the significance of diazepam effects mediated via hCAR in human hepatocytes nor the correlation of its effects between rodent and human models have been examined. In the human liver, diazepam is mainly metabolized by the CYP3A4 and CYP2C19 enzymes, during which both genes are targets of activated CAR [9,10,11]. It has also been reported that inhibition of CYP2B6, which is the most typical CAR inducible cytochrome P450 enzyme, decreases metabolism of diazepam [12]. Therefore, diazepam-mediated hCAR activation should be also considered to elucidate its own feedback metabolism regulation.

Today, we have no efficient hCAR ligand that might help us define the value of CAR in human therapy. For instance, 6-(4-chlorophenyl) imidazo [2,1-b][1,3]thiazole-5-carbaldehyde-O-(3,4-dichlorobenzy-l)oxime (CITCO) is the only specific human ligand available for experimental purposes [13]. The discovery of a hCAR activator suitable for clinical studies or one already used in pharmacotherapy would be a milestone in the characterization of hCAR as a molecular target in pharmacotherapy. Among the variety of compounds characterized as hCAR activators using in vitro high-throughput screenings, only a few compounds are on the market [7,14,15]. For instance, the routinely used pharmaceuticals diazepam and phenytoin have been described as hCAR activators in vitro, but only one solitary drug-to-drug interaction possibly connected with hCAR activation has been reported [16].

Phenobarbital is an indirect and nonspecific activator of both hCAR and rodent CAR, but also the pregnane X receptor (PXR). Studies with phenobarbital indicate that the mechanism of liver tumor promotion connected with CAR activation is relevant only for rodent models and not for humans [17]. Consequently, it is not always possible to extrapolate data obtained in animal models to hCAR and clinical situations in humans.

hCAR displays a high degree of constitutive transcriptional activity. Thus, numerous hCAR assays have not been sensitive, or they have provided biased data in the identification of novel hCAR ligands. In the past 10 years, new efficient methods for CAR activation testing have been developed, including translocation assays and gene reporter assays with different hCAR variants or mutants [7,18,19]. In addition, advanced tools and methods in the study of specific gene expression after hCAR activation have been developed such as HepaRG CAR-knockout cells and mice with humanized CAR [20].

In this report, we used different molecular biology methods to characterize hCAR activation by diazepam and its metabolites, its mechanism, and the significance of these events in vivo. Employing a translocation assay, gene reporter assays, in silico docking, a TR-FRET assay, as well as induction studies in primary human hepatocytes (PHHs), HepaRG cell lines, and in vivo in CAR humanized mice, we characterized diazepam as a novel direct hCAR in vitro ligand, but with no significant in vivo effects in CAR humanized mice in therapeutically relevant doses.

## 2. Materials and Methods

### 2.1. Chemicals

Diazepam (D0899), oxazepam (O5254), temazepam (T8275), and desmethyldiazepam (D7282) were obtained from Sigma-Aldrich (St. Louis, MO, USA), which is now known as Merck (Darmstadt, Germany). Control compounds CITCO (6-(4-chlorophenyl) imidazo [2,1-b][1,3]thiazole-5-carbaldehyde-O-(3,4-dichlorobenzy-l)oxime), rifampicin (RIF), and PK11195 were also purchased from Sigma-Aldrich. Phenobarbital (Luminal^®^ 200 mg/mL injection) was manufactured by Desitin Pharma spol.s.r.o. (Prague, Czech Republic).

### 2.2. Cell Culture

COS-1 (SV40 transformed African green monkey kidney) and human hepatocellular carcinoma HepG2 were purchased from the European Collection of Authenticated Cell Cultures (Salisbury, UK) and cultured at 37 °C in a 5% CO_2_ atmosphere in antibiotic-free Dulbecco’s Modified Eagle Medium(DMEM medium (Invitrogen, Carlsbad, CA, USA) supplemented 10% fetal bovine serum (FBS, Sigma-Aldrich) and sodium-pyruvate (Sigma-Aldrich).

### 2.3. Translocation Assay

COS-1 cells were seeded on 48-well plates (20,000 cells/well) and, 24 h after seeding, were transfected (Lipofectamine™ 3000) with 100 ng/well pEGFP-hCAR+Ala construct. The cells were treated with CITCO (10 μM), diazepam, nordazepam, temazepam and oxazepam (10 μM and 30 μM), or control (DMSO 0.1%) for 24 h. For microscopy, living cells were stained with Hoechst 33342 (0.2 µM, 5 min at 37 °C) (Sigma-Aldrich). Confocal microscopy was performed with a Nicon Ti ECLIPSE microscope and Nikon A1 plus camera (Nikon, Tokyo, Japan) using 405 and 488 nm lasers. Microphotographs were taken using the NIS Elements AR 4.20 software (Laboratory Imaging, Czech Republic). Four microphotographs of every treatment were taken and cells with green fluorescence protein (GFP) cytosolic or nuclear localization was counted in more than 150 cells in each picture. Each experiment was performed in biological triplicates (*n* = 3).

pEGFP-hCAR construct kindly donated by Dr. Y. Kanno [21] was used for site directed mutagenesis (using GeneArtTM Site-Directed Mutagenesis System, Thermo Fisher Scientific, Waltham, MA, USA). To insert extra alanine at position 271 of the CAR-LBD, the primers were used as previously described (50-CCCTCTTCTCTCCTGCTGACCGACCTGGAGTTAC-30 and 50-GTAACTCCAGGTCGGTCAGCAGGAGAGAAGAGGG-30) [18].

### 2.4. Docking

#### 2.4.1. Receptor and Ligands Preparation

The crystal structure of the hCAR model was retrieved from the RCSB Protein Data Bank (www.rcsb.org) (PDB code: 1XVP) [22]. The model was limited to chains D (containing the ligand binding domain) and H (nuclear receptor coactivator). The binding domain was checked for possible errors and clashes and then prepared by standard procedure employing AutoDock Tools 1.5.6 [23]. Water molecules were deleted, polar hydrogens were added, Kollman charges were assigned, and the prepared model was converted to a PDBQT format.

Structures of ligand molecules were drawn in PerkinElmer Chem3D (version 19.0.1.28, Waltham, MA, USA), energetically minimized utilizing the inbuilt MM2 force filed and exported as PDB files. Preparation of ligands was done by AutoDock Tools 1.5.6. Gasteiger charges were computed, non-polar hydrogens were merged, the torsion tree was built, and, finally, the model was exported as a PDBQT file. The target grid box defining the CARs active site was defined by a box with side lengths of 22 × 25 × 22 grid points (1 Å spacing) and centered at (24, 53, 30).

#### 2.4.2. Molecular Docking

Docking experiments were performed with diazepam, its three metabolites (nordazepam, oxazepam, and temazepam) and originally bound ligand CITCO employing AutoDock Vina 1.1.2. [24]. The exhaustiveness was increased to 16, the number of binding modes was limited to 5, and, otherwise, the default values were kept for the rest of the parameters. Five independent runs with random seed were performed for each ligand. Average affinity for corresponding poses is presented as the final affinity value.

#### 2.4.3. Figures

Figures representing the docking results were generated in Chimera 1.13 [25]. Only residues in proximity to ligands are displayed. All other residues and all non-polar hydrogens are omitted for clarity. The 2D ligand-protein interaction diagram was generated in LigPlot+ [26].

Numbering in figures and in text is fully in accordance with the numbering used in the 1XVP model. Nevertheless, if this sequence is compared with the canonical sequence from Uniprot.org databank (Q14994-1), residues 232–235 are missing and, therefore, numbering differs by 4 from this point.

### 2.5. Time-Resolved Fluorescence Energy Transfer (TR-FRET CAR) Coactivator Binding Assay

The LanthaScreen^®^ TR-FRET CAR Coactivator Binding Assay (Thermo Fisher Scientific, Waltham, MA, USA) was used with some modifications to the manufacturers’ protocol reported in our previous paper [27]. In order to allow better interaction of benzodiazepines with the CAR LBD, incubation time was extended to 3 h. For fluorescence measurement, the Synergy Biotek plate reader was used with appropriate filters (BioTek Instruments Inc., Winooski, VT, USA). Results were calculated as an interaction of the CAR-LBD with the coactivator fragment relative to the control (DMSO 0.1%). Data are presented as the means and SD of a technical quadruplicate (*n* = 4).

### 2.6. Plasmids

CYP2B6-luc reporter plasmid (originally entitled as B-1.6k/PB/XREM) was kindly donated by Dr. Hongbing Wang (University of Maryland School of Pharmacy, Baltimore, MD, USA). The expression construct for a ligand-activated CAR receptor transcription variant 3 (pTracer-CMV2-CAR3) was a kind gift from Dr. C. J. Omiecinski (Pennsylvania State University, State College, PA, USA). Another expression vector for CAR variant 3 (pcDNA3.1-CAR3, Clone ID: OHu09315D, Accession No. NM_005122.4) used for data presented in Figure 2B was obtained from Genscript (NJ, USA). Mutant variants Il164S, L206S, and L242S of the CAR3 were synthetized by Genscript from the pcDNA3.1-CAR3 vector. The expression plasmid pSG5-hRXRα was a generous gift from Dr. C. Carlberg (University of Kuopio, Kuopio, Finland).

CAR-LBD assembly assays were performed with constructs encoding C (151–349 aa, helices 3–12) and N (103–150 aa, helix 1) terminal parts of hCAR using the protocol described in our previous report [27]. pGL5-luc and pRL-TK were obtained from Promega (Hercules, CA, USA).

pGAL4-CAR+AAA construct encoding for human CAR-LBD with three extra alanine residues was a gift from Dr. Albert Braeuning (BfR Institute, Berlin, Germany).

### 2.7. Transient Transfection and Luciferase Gene Reporter Assays

All transient transfection gene reporter assays were carried out using Lipofectamine™ 3000 (Thermo Fischer Scientific, Waltham, MA, USA), according to the manufacturer’s protocol. The HepG2 or COS-1 cells were seeded at density 40,000 cells/cm^2^ on 48-well plates and were transfected after 24 h with either CAR3 variant expression vector and heterodimerization partner RXRα expression vectors (100 ng/well) and the p2B6-luc luciferase reporter construct (150 ng/well). In CAR3-LBD mutant assays CAR3 vectors were used with mutated amino acids in ligand binding cavity, such as Il164S, L206S, and L242S. In the CAR assembly assay, cells were transfected with two constructs encoding two parts of the CAR ligand binding domain, C (151–349 aa, helices 3–12) and N (103–150 aa, helix 1) (100 ng/well of each) together with the pGL5-luc (150 ng/well) luciferase reporter plasmid (Promega, Hercules, CA, USA) containing an upstream activation domain (UAS) binding domain. In the mammalian two-hybrid assay, the fusion plasmid GAL4-CAR+AAA (100 ng/well) was co-transfected together with the VP16-SRC-1 receptor interacting domain (100 ng/well), heterodimerization partner RXRα (50 ng/well), and pGL5-luc (150 ng/well) luciferase vector. All transient transfection assays were normalized with the *Renilla reniformis* luciferase transfection control plasmid (pRL-TK, 30 ng/well). After 24 h from transfection cells, they were treated with tested compounds for the following 24 h. Luminescence activity was measured using the Dual luciferase detection kit (Promega) and fold-activity was expressed relative to the vehicle-treated samples (0.1% DMSO). In all experiments, results are presented as means and SD from at least three independent experiments performed in triplicates, except for COS-1 CAR assembly assay, where representative experiments are depicted.

### 2.8. qRT-PCR

Cryopreserved differentiated HepaRG™ cells (HPRGC10, ThermoFisher Scientific, Waltham, MA, USA) were seeded according to the manufacturer’s protocol and cells were treated by studied compounds after 72 h of stabilization (control (DMSO 0.1%), CITCO 10 µM, diazepam 30 and 50 µM, nordazepam, temazepam, and oxazepam 50 µM, respectively) for 48 h. Total RNA was isolated using TRIZOL^®^ and reverse transcription was performed with the RevertAid RT Reverse Transcription Kit. qPCR was performed using TaqMan™ Fast Advanced Master Mix with TaqMan probes for *CYP2B6* (Hs04183483_g1), *GAPDH* (Hs02786624_g1), and *B2M* (Hs00187842_m1) genes (all reagents for qRT-PCR were obtained from Thermo Fischer Scientific Catalog number: 4331182, Waltham, MA, USA). The delta-delta method was used for gene expression quantification normalized to *GAPDH* and *B2M* gene expression average. Three technical replicates were used for each reaction.

### 2.9. Primary Human Hepatocyte Isolation and Culture

Primary human hepatocytes (PHHs) were isolated as described previously [28] from liver resections performed in adult patients for medical reasons unrelated to our research program. Liver samples were obtained from the Biologic Resource Center of Montpellier University Hospital (CRB-CHUM, http://www.chumontpellier.fr, Biobank ID: BB-0033-00031), and this study benefitted from the expertise of Dr. Benjamin Rivière (hepatogastroenterology sample collection) and Dr. Edouard Tuaillon (CRB-CHUM manager). The patients’ clinical characteristics are the following: Liv1—female, 28 years, focal nodular hyperplasia, Liv2—female, 45 years, adenoma. The procedure was approved by the French Ethics Committee and written or oral consent was obtained from the patients.

PHHs were seeded at confluency (2.1 × 10^5^ cell/cm^2^) in ISOM medium supplemented with 2% fetal bovine serum and cultured in 5% CO^2^ humidified atmosphere at 37 °C. ISOM medium was changed to hepatocyte growth medium (HGM: WME medium supplemented with 5 µg/mL insulin, 0.1 µM hydrocortisone, 10 µg/mL transferrin, 250 µg/mL ascorbic acid, 3.75 mg/mL fatty acid-free bovine serum albumin, 2 mM glutamine, penicillin, and streptomycin) at day 1 post-seeding. One day after, the cells were treated with tested compounds for 24 h.

#### 2.9.1. siRNA Transfection

Adherent PHHs were transfected with 20 nM non-targeting siRNA (scrambled, siSC) or siRNAs specific for CAR or Pregnane-X receptor (PXR, siCAR or siPXR, Dharmacon, Lafayette, CO, USA) at day 1 and day 3 after seeding using Lipofectamine RNAiMAX reagent (Life Technologies, Carlsbad, CA, USA). At day 4 post-seeding, PHHs were treated with tested molecules for 24 h.

#### 2.9.2. RNA Isolation and RT-PCR Assays of PHHs

After extraction with Trizol reagent (Life Technologies), 500 ng of total RNA was reverse-transcribed using a random hexaprimer and the MMLV Reverse Transcriptase Kit (Life Technologies). Quantitative polymerase chain reactions were performed using the Roche SYBER Green reagent and a LightCycler 480 apparatus (Roche Diagnostic, Meylan, France). The amplification specificity was evaluated by determining the product melting curve. Results are expressed as indicated in the figure legends. The primer sequences summarized are the following: *CYP2B6*, ATGGGGCACTGAAAAAGACTGA, AGAGGCGGGGACACTGAATGAC, *GADPH*, GTCTCCTCTGACTTCAACAGCG, ACCACCCTGTTGCTGTAGCCAA, respectively. The following program was used: one step at 95 °C for 10 min and then 50 cycles of denaturation at 95 °C for 10 s, annealing at 62 °C for 15 s and elongation at 72 °C for 15 s.

### 2.10. Animal Experiments

All animal studies were performed in accordance with European Directive 86/609/EEC and they were approved by the Czech Central Commission for Animal Welfare. Humanized PXR-CAR-CYP3A4/3A7 mice (model 11585) were obtained from Taconic (Rensselaer, NY, USA) and kept in a temperature-controlled and light-controlled facility with a 12-h light-dark cycling. All animals had free access to a commercially available laboratory chow diet (Velaz, Prague, Czech Republic). Male 9–17 weeks old animals were randomized into three groups (control (vehicle, PEG300 and H_2_O 1:1, *n* = 4), CITCO (10 mg/kg, *n* = 3), diazepam (1 mg/kg, *n* = 6)), and formulations were administered in two following days intraperitoneally. Animals were sacrificed 24 h after the second administration and livers were removed and snap frozen in liquid nitrogen for further total RNA isolation. To detect mRNA levels, the same procedure was performed as in cellular experiments and TaqMan specific probes (from Thermo Fischer Scientific Catalog number: 4331182) for *Cyp2b10* (Mm00456588_mH), *Cyp2c29* (Mm00725580_s1), *CYP3A4* (Hs00604506_m1), *Fasn* (Mm00662319_m1), *G6pc* (Mm00839363_m1), *Mki67* (Mm01278617_m1), *Foxm1* (Mm00514924_m1), *Gadd45b* (Mm00435123_m1), *Pcna* (Mm00448100_g1), *Gapdh* (Mm99999915_g1), and *B2m* (Mm00437762_m1) genes were used.

### 2.11. Statistics

Data are presented as the mean ± standard deviation (SD). A one-way analysis of variance (ANOVA) with a Dunnett’s post hoc test or Bonferroni test were applied. GraphPad Prism 8.4.3 Software (GraphPad Software, Inc., San Diego, CA, USA) was used to perform statistical analysis. A *p*-value of < 0.05 was considered to be statistically significant.

## 3. Results

### 3.1. Diazepam and its Metabolites Nordazepam, Temazepam, and Oxazepam Significantly Translocate EGFP-hCAR+Ala into the Nucleus

**Figure 1:**
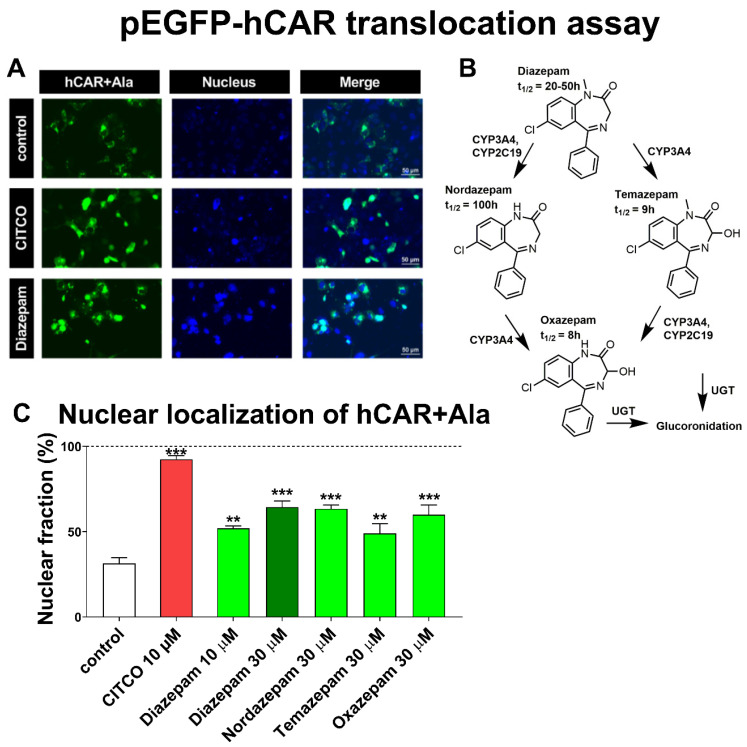
Diazepam, nordazepam, temazepam, and oxazepam significantly translocate EGFP-CAR+Ala chimera in COS-1 cells. (**A**) Photomicrographs of fluorescently-labeled CAR+Ala in translocation assays in living COS-1 cells treated with CITCO or diazepam (both at 10 μM) for 24 h. Nuclei were stained with Hoechst33342 (0.2 µM for 5 min). (**B**) The scheme of diazepam metabolism to nordazepam, temazepam, and oxazepam. (**C**) Quantitative presentation of nuclear localization of EGFP-CAR+Ala protein after treatment with diazepam and its metabolites. COS-1 cells were transfected with the pEGFP-CAR+Ala construct 24 h after seeding and after overnight stabilization, cells were treated for the following 24 h with diazepam (10 μM or 30 μM), nordazepam, temazepam, or oxazepam (30 μM) or with the CAR agonist CITCO (10 μM). Nuclear translocation is depicted as a percentage of cells with complete nuclear EGFP localization of expressed EGFP-CAR+Ala. Data are presented as the means ± SD from three independent experiments (*n* = 3). ** *p* < 0.01, *** *p* < 0.001, statistically significant effects of tested benzodiazepines or CITCO to control cells.

Diazepam has been shown as a strong translocating agent with the Ad/EYFP-hCAR vector in primary human hepatocytes [7]. We sought to determine if diazepam and its metabolites promote the nuclear translocation of the EGFP-CAR+Ala mutant in COS-1 cells. This CAR mutant displays reduced basal constitutive activity and can be significantly translocated by a ligand-mediated CAR activation into the nucleus [18]. In addition, this method can be used for transient transfection in an immortalized cell line without metabolic biotransformation capacity (such as COS-1 cells) in a high-throughput manner, even though there is higher basal nuclear localization of the CAR+Ala chimeric protein (31.3%, Figure 1A,C) in comparison to the primary hepatocyte model.

**Figure 2:**
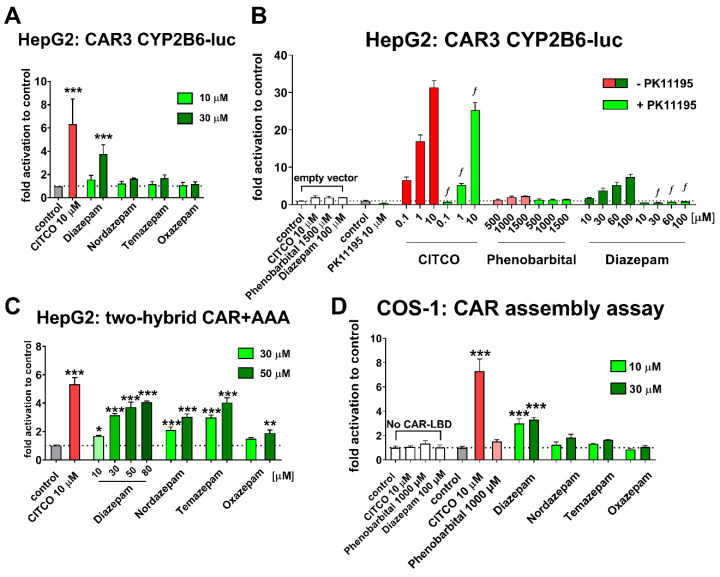
Diazepam, nordazepam, temazepam, and oxazepam activities in hCAR-responsive luciferase gene reporter assays. (**A**) The pTracer-CMV2-CAR3 variant expression construct was used in luciferase gene reporter assay in HepG2 cells, (**B**) and the pcDNA3.1-CAR3 expression vector was used in luciferase reporter assays on the inhibition with the inverse agonist PK11195 after activation with CITCO or diazepam. (**C**) The mammalian two hybrid assay of CAR+AAA LBD-SRC1 interaction in HepG2 cells. (**D**) The CAR-LBD assembly assay was performed in COS-1 cells treated with diazepam and its metabolites. Luciferase reporter gene assays were performed in HepG2 or COS-1 cells transiently transfected with either human CAR3 variant expression vector and RXRα construct with CAR-N and CAR-C plasmids or pGAL4-CAR+AAA and VP16-SRC1 constructs and an appropriate responsive luciferase reporter promoter constructs (p2B6-luc or pGL5-luc) together with *Renilla* expression construct for transfection normalization (see Section 2). To exclude nonspecific activation of the p2B6-luc or pGL5-luc luciferase reporter constructs, the appropriate empty pTracer-CMV2 vector or mock transfection were used (Figure 2B,D). Cells were treated with diazepam, nordazepam, temazepam, or oxazepam (10 μM or 30 μM) together with CAR direct agonist CITCO (10 μM) and CAR indirect activator phenobarbital (500 µM, 1000 µM, and 1500 µM) for 24 h after transfection. In the CAR3 inhibition study (**B**), cells were also co-treated with PK11195 (10 μM) together with diazepam (from 10 μM to 100 μM), phenobarbital (500, 1000, and 1000 μM) and CITCO (from 0.1 μM to 10 μM) for 24 h. Relative activation is depicted as relative fold activation to control the samples (DMSO 0.1%) and data are presented as the means ± SD from three independent experiments (*n* = 3) performed in triplicates or as a representative experiment (**C**). * *p < 0.05*, ** *p* < 0.01, *** *p* < 0.001, statistically significant effects of CITCO or diazepam to control the cells. *f* *p* < 0.05. Statistically significant effect of PK11195 inhibitor on CAR3 activation.

We observed significant translocation of the chimeric EGFP-CAR+Ala protein mediated by diazepam (52% at 10 µM and 65% at 30 µM, respectively) and by the prototype high-affinity ligand CITCO (93% at 10 µM), respectively (Figure 1A). Since diazepam is metabolized into its metabolites including nordazepam, temazepam, and oxazepam in the liver, and all these metabolites have a long half-life (Figure 2B), we also tested if these metabolites promote EGFP-CAR+Ala translocation. Nordazepam and oxazepam had a similar effect as diazepam at 30 µM in the translocation assay. Temazepam promoted was less pronounced, but still has a significant translocation (49% at 30 µM) (Figure 1C).

### 3.2. Diazepam is a Direct hCAR Activator in Luciferase Reporter Gene Assays and Loses Activity after Metabolic Oxidation

In the following set of experiments, we sought to determine if diazepam and its metabolites, known as nordazepam, temazepam, and oxazepam, interact with hCAR-LBD in luciferase gene reporter assays. Diazepam was characterized as an indirect CAR activator based on the experiments with wild type hCAR variant with high constitutive activity and on the absence of competition between diazepam and the CAR inhibitor PK11195 [7]. To verify this hypothesis, we used the pTracer-CMV2-CAR3 variant expression vector in luciferase gene reporter system in HepG2 cells, which has a high ligand-activated response with low constitutive activity. As shown in Figure 2A, diazepam was able to significantly activate hCAR variant 3 both at 10 μM and 30 μM concentration. However, its metabolites do not display any activities and we observed no significant response in these experiments. To further study the mechanism of hCAR activation with diazepam, we used the CAR inverse agonist PK11195 [29] and the commercial hCAR variant 3 expression construct pcDNA3.1-CAR3. Co-treatment with PK11195 in pcDNA3.1-CAR3 reporter assays leads to a significant reduction in CAR3 activation with diazepam, suggesting competitive inhibition in hCAR binding cavity (Figure 2B). Diazepam showed the same dose-response activation and inhibition as the model direct activator CITCO [13]. However, the prototypical indirect activator phenobarbital was not able to interact with CAR3 and compete with PK11195 in the experiments. Unfortunately, we did not reach a plateau of maximum activation in these assays to calculate EC_50_ for diazepam. In the similar experiments, when we replaced the CAR3 expression vector with an empty pTracer-CMV2 vector, we did not observe any significant activation with CITCO (10 μM), phenobarbital (1500 μM), or diazepam (100 μM), respectively (Figure 2B).

Subsequently, we used a mammalian two-hybrid assay to test whether diazepam and its metabolites promotes the interaction between CAR-LBD and the SRC-1 coactivator. In these experiments, we used highly the inducible variant CAR+AAA LBD expression construct. In two-hybrid assay (Figure 2C), diazepam showed significant dose-dependent activity from 10 µM up to 80 µM. Similarly, nordazepam and temazepam promoted a significant interaction at 30 µM and 50 µM, respectively. Oxazepam was significantly active only at 50 µM. To further corroborate a direct hCAR activation by diazepam, we performed the CAR-LBD assembly assay in COS-1 cells (Figure 2D). In the CAR assembly assay, two fragments of CAR-LBD fold together around a direct ligand. Therefore, this assay is highly sensitive to distinguish between direct and indirect activation. In the assembly assay, only diazepam and CITCO significantly activate luciferase reporter gene expression in the same manner as in the CAR3 reporter assay (Figure 2A,D). The indirect activator phenobarbital did not activate this assay. In addition, neither CITCO, phenobarbital, nor diazepam activated the pGL5-luc reporter construct in the absence of CAR LBD vectors (Figure 2D).

### 3.3. Diazepam Interacts with CAR-LBD Protein in TR-FRET Assay in the Absence of a Cellular Background

**Figure 3:**
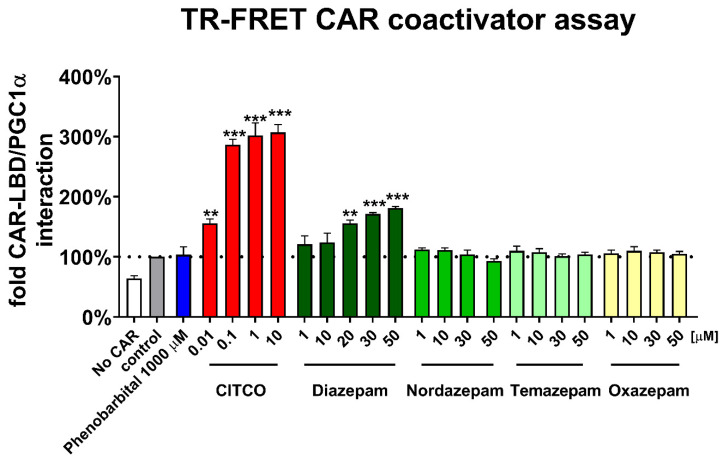
TR-FRET CAR coactivator assay. LanthaScreen^®^ TR-FRET coactivator binding assay was utilized to determine interactions of tested compounds (diazepam, nordazepam, temazepam, and oxazepam) with the recombinant CAR-LBD. The manufacturer’s protocol was followed with modifications described in the Section 2. Data are presented as the relative interaction between CAR-LBD and PGC1α fragment to the control (0.1% DMSO) sample (as a percentage). Data are presented as the mean ± SD from three assays performed in quadruplicate. ** *p* < 0.01, *** *p* < 0.001, statistically significant effects of CITCO or diazepam to control samples.

Subsequently, we studied the behavior of these compounds in a TR-FRET CAR coactivator assay with a recombinant hCAR LBD protein. This assay allows detection of the molecule-to-CAR LBD protein interaction when the cellular background and signaling is avoided. Thus, indirect CAR activation via dephosphorylation is not possible in the protein assay (Figure 3). In the TR-FRET CAR coactivator assay, diazepam has shown statistically significant activity from 20 μM. Other benzodiazepines had no statistically significant activity to activate CAR-LBD in the assay up to 50 μM (Figure 3). Phenobarbital did not activate the CAR LBD in the assay. Our TR-FRET assay data correspond with results from the luciferase gene reporter assays (Figure 2) and confirm direct CAR-LBD activation with diazepam, but not with temazepam, oxazepam, or nordazepam. Consequently, because diazepam reached incomplete CAR activation compared to the high-affinity agonist CITCO, we may characterize diazepam as a partial agonist of hCAR.

### 3.4. Diazepam and Its Metabolites Fits hCAR-LBD Cavity in Silico Docking Experiments

**Figure 4:**
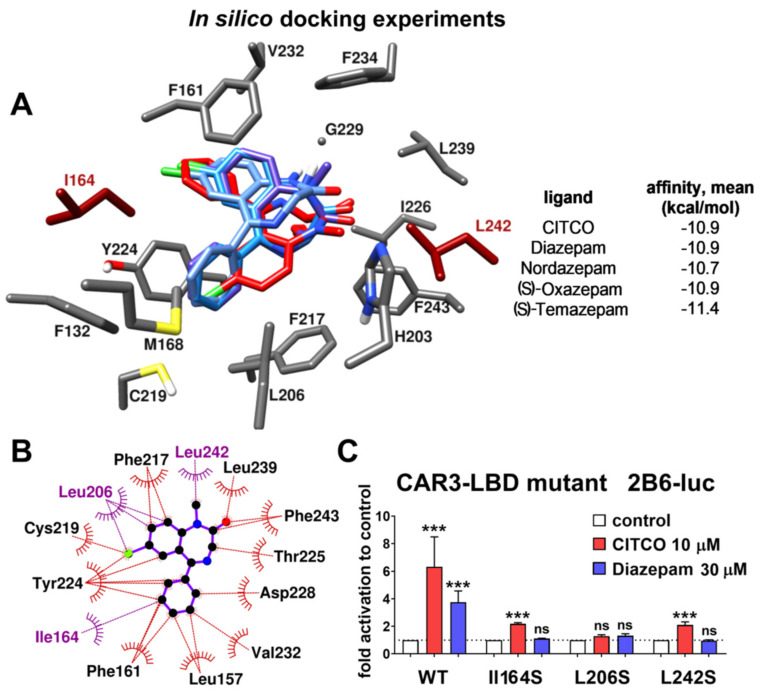
Diazepam and its metabolites pose within the hCAR LBD as determined in in silico docking experiments. (**A**) Positioning of diazepam (red) and its metabolites (blue) docked in hCAR LBD with their affinities to the receptor. (**B**) Scheme of diazepam interactions with hCAR LBD cavity. (**C**) Effects of CAR3 with mutated amino acids Ile164Ser, Leu206Ser, and Leu242Ser in luciferase reporter gene reporter assays. Docking was performed in AutoDock Vina and illustrations were generated in Chimera and LigPlot+. Luciferase gene reporter assays have been performed with the same setup as experiments depicted in Figure 2A with use of the CAR3 wild-type and CAR3 mutant vectors. *** *p* < 0.001, statistically significant effects of CITCO or diazepam to control cells transfected with the appropriate expression vectors.

The original ligand CITCO was redocked and compared with two proposed poses presented by Xu, Lambert, Wisely, Warren, Weinert, Waitt, Williams, Collins, Moore, Willson and Moore [22]. RMSD for heavy atoms was 0.56 and 1.39 Å, respectively. The studied benzodiazepines and the ligand binding domain (Figure 4A,B) are apolar. Therefore, hydrophobic interactions are the driving force. Benzodiazepines made the closest hydrophobic contacts with Ile164, Leu206, and Val232. Other prominent amino acids in the CAR-LBD important for tested benzodiazepines binding are Phe132, Leu157, Phe161, Phe217, Leu242, and Phe243. The position of all ligands was further stabilized by π-interaction between their 5-phenyl C-ring and Tyr224. Hydrogen bonds were observed only for (S)-Oxazepam and (S)-Temazepam between their 3-hydroxyl group and Thr225 backbone oxygen. This might explain the better binding affinity values for (S) over (R) enantiomers for these two ligands (−10.9 kcal/mol compared to −10.4 kcal/mol for oxazepam and −11.4 kcal/mol and compared to −10.4 kcal/mol for temazepam, respectively). Although CITCO and benzodiazepines show different activities toward hCAR (Figure 1, Figure 2 and Figure 3), their binding activities are comparable in the docking experiment (Figure 4A). This is in line with known promiscuity of the CAR-LBD and suggests that the hCAR cavity can accommodate benzodiazepine molecules. Unfortunately, docking assays are not able to evaluate in detail affinities of smaller molecules interacting exclusively via hydrophobic interactions with the cavity.

Next, we verified the proposed positioning of these compounds in the CAR-LBD by preparing mutants Ile164Ser, Leu206Ser, and Leu242Ser in the CAR3 vector backbone to test the activity in transient luciferase reporter gene assays (Figure 4C). In the case of Leu206Ser, both CITCO and diazepam did not activate the mutated CAR3, suggesting that Leu206 is necessary for CAR3 activation by these ligands. In the case of Ile164Ser and Leu242Ser, CITCO is a significant activator as in non-mutated CAR3. However, diazepam lost activities in these mutant CAR3 variants. We, therefore, suppose that benzodiazepine molecules are stabilized in the hCAR cavity by its lipophilic surface, and all polar modifications of the cavity with serine residues in mutated vectors abolish this interaction.

### 3.5. Diazepam Promotes Induction of CYP2B6 mRNA in Primary Human Hepatocytes

**Figure 5:**
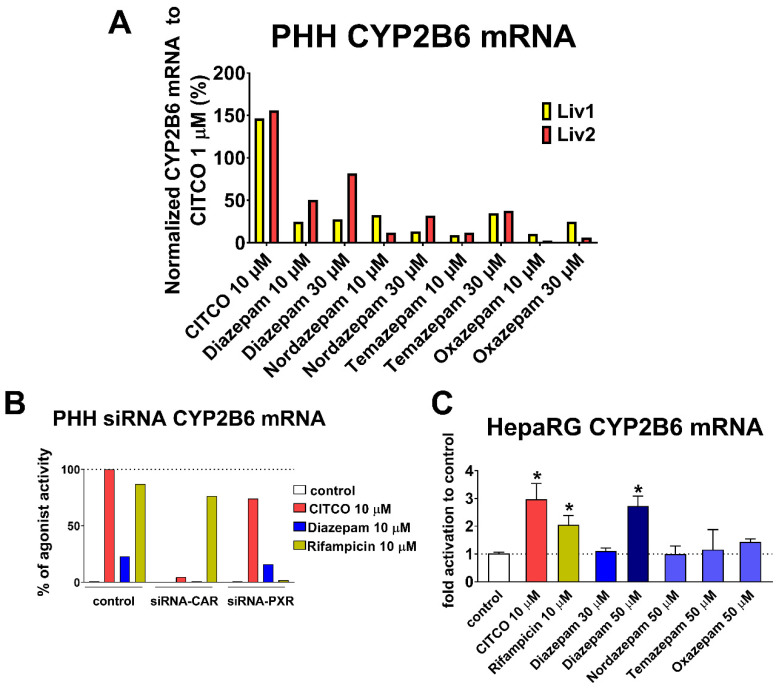
Effect of diazepam on CYP2B6 mRNA induction in hepatocyte cell lines. (**A**) PHHs from two donors treated by tested compounds. (**B)** PHHs pretreated with CAR and PXR siRNA or with scramble siRNA (control). (**C)** Differentiated HepaRG. Cells were treated with vehicle (DMSO 0.1%) in control, CITCO 10 µM, rifampicin 10 µM, and benzodiazepines at 10, 30, or 50 µM for 24 h in PHHs or for 48 h in HepaRG cells. CYP2B6 mRNA expression levels were analyzed by RT-qPCR. CYP2B6 induction is shown relative to CITCO 1 µM (100%) in PHHs (**A**), relative to CITCO 10 μM (100%) in PHH (**B**) or as fold activation to control (set to be 1) in HepaRG cells **(C)** and data are presented as a mean of technical triplicate. * *p* < 0.05, statistically significant effects of treatment to control cells.

In the next experiment, we aimed to confirm that diazepam interacts with the CAR nuclear receptor in liver cells and promotes CYP2B6 target gene expression. Diazepam was already reported as the CYP2B6 mRNA inductor at a high concentration (50 μM) [7]. We also wanted to elucidate activities of nordazepam, temazepam, and oxazepam. In two isolations of primary human hepatocytes, CYP2B6 mRNA was induced by diazepam even at 10 µM, while activities of metabolites were lower in comparison with diazepam (Figure 5A). We showed in our recent paper that diazepam (18 μM) does not significantly activate PXR and does not significantly up-regulate CYP3A4 mRNA in PHHs [30]. To discriminate between CAR and PXR activation in CYP2B6 mRNA induction, we used siRNA to suppress the expression of CAR or PXR in PHHs. In agreement, activities of model ligands CITCO in siRNA-CAR-treated and rifampicin in siRNA-PXR-treated PHHs were abolished (Figure 5B). We also found that CYP2B6 mRNA induction mediated by diazepam was abolished after siRNA-CAR treatment, but there was almost no difference between control (scramble treatment) and siRNA-PXR treatment (Figure 5B). These results show that diazepam up-regulates CYP2B6 mRNA only via CAR activation.

To study diazepam-hCAR interaction in another cellular model with natively expressed CAR, we implemented a differentiated HepaRG cell line (Figure 5C). In HepaRG cells, diazepam had no statistically significant effect at 30 µM and induced CYP2B6 mRNA only at 50 µM. However, diazepam metabolites had no activities in HepaRG cells at 50 µM. We suppose that lower benzodiazepine activities in HepaRG cells are caused by lower CAR expression and less sensitivity of the model to CAR-mediated induction. Based on these data from the two models, we suppose that diazepam is able to activate CAR target genes regulation in vitro.

### 3.6. Diazepam Has No Effect on Xenobiotic and Endobiotic Metabolism and on Liver Proliferation Genes In Vivo in hCAR/hPXR/hCYP3A4 Mice

**Figure 6:**
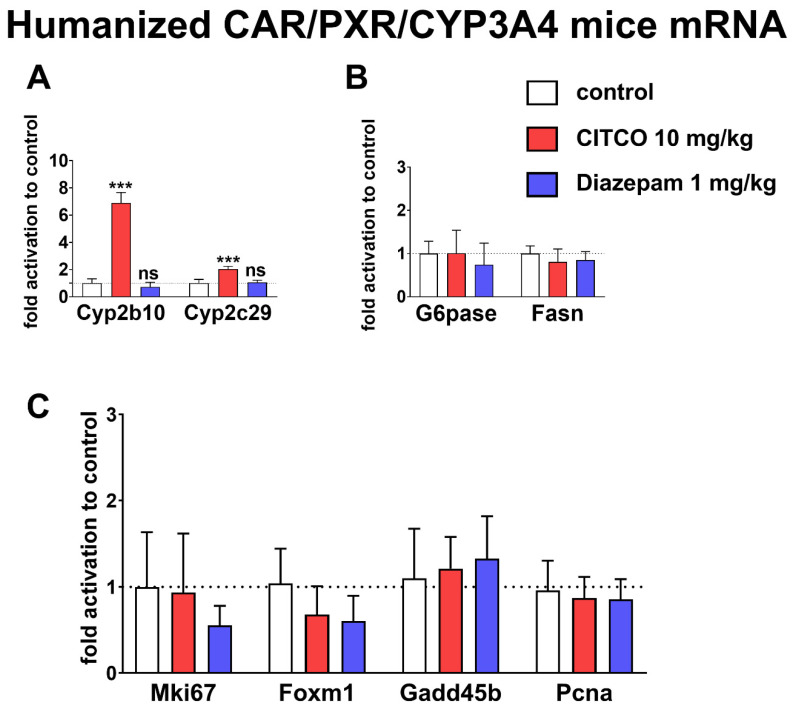
Diazepam has no effect on CAR activation in vivo in hCAR/hPXR/hCYP3A4 mice. (**A**) mRNA levels of cytochromes Cyp2b10 and Cyp2c29, (**B**) mRNA levels of *Fasn* and *G6pc* genes, and (**C**) mRNA levels of proliferation markers *Mki67*, *Foxm1*, *Gadd45b*, and *Pcna* genes. Animals were treated with the control (vehicle, PEG300:H_2_O 1:1, *n* = 4), diazepam (1 mg/kg, *n* = 6), or with CITCO (10 mg/kg, *n* = 3) with two doses in two consecutive days and sacrificed the day after. Subsequently, RNA was isolated from the liver and mRNA levels detected by qRT-PCR. Data are presented as means and SD of fold activation to control, when the delta-delta method was used for relative quantification of RT-PCR data. *** *p* < 0.001, statistically significant effect of CITCO.

Because diazepam was characterized as a rodent Cyp2b10 inducer (analog of human CYP2B6) and it even increased liver weight and promoted liver tumorigenesis (Parkinson 2006), we aimed to study the influence of diazepam in vivo in hCAR/hPXR/hCYP3A mice. CITCO (at a dose of 10 mg/kg) significantly upregulated CAR inducible cytochromes P450 genes *Cyp2b10* and *Cyp2c29* mRNA (Figure 6A). Diazepam (at a dose of 1 mg/kg) stimulated no up-regulation on the hCAR regulated genes *Cyp2b10* and *Cyp2c29* mRNA (Figure 6A). Furthermore, we analyzed the effect of hCAR activation on key enzymes in lipogenesis (Fasn) and gluconeogenesis (G6pc) after treatment with diazepam and CITCO (Figure 6B). Finally, we looked at genes connected to liver proliferation and non-genotoxic tumorigenesis via Car activation in rodents, such as *Mki67*, *Foxm1*, *Gadd45b*, and *Pcna* (Figure 6C). There was no significant regulation of these genes by either CITCO or diazepam in these animal experiments. Therefore, we expect no in vivo effects of diazepam in therapeutic doses after a single administration on xenobiotic metabolism, lipid and glucose metabolism, and liver proliferation genes expression.

## 4. Discussion

In this report, we characterized diazepam and its metabolites nordazepam, temazepam, and oxazepam as stimulators of hCAR nuclear translocation, which is the first key step in CAR activation. We also utilized in silico docking experiments and found that these benzodiazepines fit the hCAR LBD cavity and their lipophilic moieties form non-polar interactions with the cavity. However, in luciferase gene reporter and TR-FRET assays, diazepam proved as a significant direct hCAR activator, but its metabolites display much lower activities toward hCAR. Furthermore, gene expression studies in primary human hepatocytes have shown that diazepam activates CAR, but not PXR, in the regulation of CYP2B6 mRNA. To elucidate clinical importance of this interaction, we also performed in vivo experiments with humanized CAR/PXR/CYP3A4 mice with a therapeutic-relevant dose of diazepam, and we observed no effects of diazepam on several key genes involved in xenobiotic and endobiotic metabolism and liver proliferation, which are also sensitive target genes to CAR activation.

Diazepam was identified as the translocator of wild-type EYFP-tagged hCAR in primary human hepatocytes [7]. However, most inducible hCAR target gene CYP2B6 mRNA up-regulation in primary human hepatocytes depends on treatment concentration, as Li, Chen, Cottrell and Wang [7] described diazepam as an inductor of both CYP3A4 and CYP2B6 mRNA at 50 µM, whereas Vrzal, Kubesova, Pavek and Dvorak [30] observed no significant induction of CYP3A4 mRNA in human hepatocytes at 18 µM. Consistently, we observed the diazepam-mediated stimulation of hCAR+Ala mutant translocation in the immortalized kidney cell line COS-1 at 10 μM and a similar stimulation by the diazepam metabolites temazepam, oxazepam, and nordazepam at 30 μM (Figure 1). Similarly, in a two-hybrid CAR+AAA LBD-SRC1 coactivator assay, diazepam, nordazepam, and temazepam were significantly active at 30 µM, while the final metabolite oxazepam had significant activity toward CAR+AAA LBD activation only at 50 µM (Figure 2C). However, in luciferase gene reporter assays with either CAR3 variant expression vector as well as in a CAR-LBD assembly assay, only diazepam significantly activated hCAR (Figure 2A,D). The CAR3 transcription variant (wild type hCAR with five extra amino acids, APYLT) and CAR+Ala differs in their activities after ligand activation, when artificial CAR+Ala shows higher capacity for activation when compared to the CAR3 variant [18]. Thus, the activation of EGFP-CAR+Ala and pGAL4-CAR+AAA by all tested benzodiazepines, but only diazepam-mediated activation of CAR3 gene reporter and the CAR assembly assay is the consequence of differently inducible CAR variants.

In contrast to primary human hepatocytes, both COS-1 and HepG2 cell lines lack any reasonable metabolic activity to convert diazepam into its metabolites. These data, therefore, suggest that the significant translocation of hCAR and induction of CYP3A4 and CYP2B6 mRNA in primary human hepatocytes reported by Li, Chen, Cottrell and Wang [7] is not a consequence of a diazepam metabolite that can activate hCAR.

Diazepam has been proposed as a phenobarbital-like indirect CAR activator, as diazepam was not able to reverse the inhibition of the wild-type hCAR mediated by the PK11195 inhibitor in luciferase reporter assays. In our experiments with the highly ligand-activable variant CAR3 and CAR inhibitor PK11195, however, a significant effect of the inhibitor was shown on the diazepam-mediated activation of CAR3 (Figure 2B). In agreement with this finding, we observed that diazepam activates the luciferase reporter in the CAR-LBD assembly assay (Figure 2D). In neither CAR3 reporter nor assembly luciferase assays, the indirect CAR activator phenobarbital proved any interaction with CAR constructs, which confirms ability of these assays to distinguish direct CAR activation. Diazepam also promotes interaction of CAR-LBD with a coactivator peptide in the TR-FRET assay, suggesting that diazepam directly activates hCAR and its CAR3 variant (Figure 2B,D and Figure 3). Based on in silico docking experiments and experiments with CAR3-LBD mutants in a gene reporter assay, we suggest that residues Ile164 and Leu242 might be important for the positioning of diazepam in the hCAR ligand binding pocket (Figure 4).

In our subsequent RT-qPCR experiments, CYP2B6 mRNA induction by benzodiazepines was observed in primary human hepatocytes, where metabolites lost activity toward almost inactive oxazepam (Figure 5A). Diazepam showed weak to moderate CYP2B6 mRNA induction at 10 µM and 30 µM. Therefore, lower affinity and activity characterize diazepam as an hCAR low-affinity partial agonist. Diazepam also induced CYP2B6 mRNA in differentiated HepaRG cells only at the concentration of 50 µM (Figure 5C), where CAR transcriptional activity is lower compared to PPHs.

In rodent models, diazepam stimulates the induction of Cyp2b enzymes via CAR and even promotes hepatic proliferation in mice receiving high doses of diazepam [8]. Cyp2b induction and liver proliferation were for a long time considered as connected events in CAR activation, but recent studies have shown that a common hallmark of CAR activation is the induction of CYP2B6/Cyp2b10 enzymes. Liver proliferation, however, is restricted to rodent CAR [31,32]. The hCAR/hPXR/hCYP3A4 mice strain is, so far, one of the most realistic in vivo models of hCAR, responding to specific hCAR ligands and reflecting the hCAR specific transcriptional regulation limited to the mouse genome. This model was recently used not only to study xenobiotic metabolism induction [33], but even for liver proliferation studies [32].

In humanized CAR/PXR/CYP3A4 mice, CITCO treated mice (10 mg/kg) exhibited *Cyp2b10* and *Cyp2c29* genes induction. However, we observed no Cyp2b10 mRNA (the murine CYP2B6 analog) induction after 1 mg/kg diazepam administration (Figure 6A). The endogenous metabolism genes *Fasn* and *G6pase* revealed non-significant down-regulation trends. However, these genes might be co-regulated by diazepam through a translocator protein [34] (Figure 6B). Furthermore, the proliferation and apoptosis-related genes *Mki67*, *Foxm1*, *Gadd45b*, and *Pcna* were regulated neither by diazepam nor CITCO (Figure 6C). The formerly described CAR-mediated liver proliferation genes upregulation promoted by the both diazepam and CITCO were not detected in our work, as these compounds had been used in much higher doses in previous rodent studies (diazepam up to 10,000 ppm in the powdered diet or CITCO at 50 mg/kg dose, respectively) [8,35].

After chronic administration of diazepam in human medicine, plasmatic concentrations of diazepam are in the range of 0.1–1.0 µM [36]. Matsuda, et al. [37] measured portal and systemic concentration of benzodiazepine midazolam after administration of the same dose as in our in vivo experiment (1 mg/kg). They observed that midazolam concentration immediately after oral administration was at about 3.07 µM. However, after 15 to 30 min, the drug was almost completely distributed with portal concentration under 0.3 µM. Thus, we do not expect that diazepam and its metabolites activate CAR in human medicine relevant doses.

In this work, we have comprehensively described the relationship between hCAR and diazepam, which was already postulated as an indirect hCAR activator. We found a correlation in all protein and vector-based methods as well as in silico docking experiments, and we describe diazepam as a direct hCAR ligand. However, significant CYP2B6/Cyp2B10 mRNA induction was shown only in primary human hepatocytes, but not in vivo in a humanized mice model when a therapeutically relevant dose was used. This is most likely due to the low affinity of diazepam to hCAR.
